# Circ_0027885 sponges miR-203-3p to regulate RUNX2 expression and alleviates osteoporosis progression

**DOI:** 10.1186/s12891-023-07122-1

**Published:** 2024-01-02

**Authors:** Shuhua Fang, Dingwen Cao, Zhanpo Wu, Jie Chen, Yafei Huang, Ying Shen, Zengxin Gao

**Affiliations:** 1 Department of Pharmacy, Nanjing Lishui People’s Hospital, Zhongda Hospital Lishui Branch Southeast University, Nanjing, China; 2 Department of Orthopedics, Nanjing Lishui People’s Hospital, Zhongda Hospital Lishui Branch Southeast University, Nanjing, China

**Keywords:** Circ_0027885, miR-203-3p, RUNX2, Osteoporosis, Osteogenic differentiation

## Abstract

**Background:**

Osteoporosis (OP) is a progressive metabolic disorder that is difficult to cure clinically. The molecular mechanisms of OP urgently need to be further examined. This study was designed to explore the potential function of circ_0027885 during osteogenic differentiation, as well as the systematic interactions among circ_0027885, miR-203-3p and runt-related transcription factor 2 (RUNX2).

**Methods:**

Relative levels of circ_0027885, miR-203-3p and RUNX2 were analyzed with RT-qPCR and western blotting. Alizarin red staining was performed to detect the mineralization ability under the control of circ_0027885 and miR-203-3p. Dual-luciferase reporter gene assay was conducted to examine the combination among circ_0027885, miR-203-3p and RUNX2.

**Results:**

Our research demonstrated that circ_0027885 was significantly increased during hBMSCs differentiation. Overexpression of circ_0027885 notably facilitated osteogenic differentiation and upregulated RUNX2 expression, while knockdown of circ_0027885 reversed the above results. Through prediction on bioinformatics analysis, miR-203-3p was the target binding circ_0027885, and RUNX2 was the potential target of miR-203-3p. Subsequently, these changes induced by the overexpression of circ_0027885 were reversed upon addition of miR-203-3p mimic.

**Conclusions:**

Circ_0027885 could sponge miR-203-3p to regulate RUNX2 expression and alleviate osteoporosis progression.

## Background

Osteoporosis (OP) is a group of multifactorial skeletal diseases worldwide, featured with increased bone fragility, decreased bone mass and reduced bone density [1]. The high incidence of OP is most deeply connected to aging, endocrine disorders, calcium malabsorption, genetic factors and so on [2, 3]. Indeed, OP has seriously influenced health-related quality of life, and leads to an increased risk of disability and mortality, especially for elderly and postmenopausal women [4]. Up to now, the therapeutic means for OP are restricted to physical therapy, exercise therapy and drug therapy [5]. Therefore, an in-depth investigation of molecular genetics appears to be an effective therapeutic strategy for the prevention and alleviation of OP.

Recently, the role of non-coding RNA has received extensive attention in musculoskeletal conditions [6–9]. Circular RNAs (circRNAs) belong to a group of non-coding RNAs with a stable circular structure, which can prevent exonuclease-mediated degradation [10]. Owing to their unique properties, circRNAs are involved in multiple physiological processes, including cell proliferation, migration and differentiation [11–13]. Recently, a substantial number of investigations have uncovered that circRNAs may exert vital roles in the development of OP [14, 15]. A clinical trial identified that, circ_0027885 was abnormally expressed at low levels among 20 circRNAs studied in OP patients [16]. However, little is known about how it acts in the regulation of OP. Thus, it is worth exploring the function of circ_0027885 in the pathogenesis of OP.

MicroRNAs (miRNAs; 18–25 nucleotides) are a kind of endogenous non-coding RNAs that can guide mRNA post-transcriptional silencing through transcriptional degradation or translation inhibition [17, 18]. Emerging evidence has confirmed that they are actively involved in an extended range of biological processes, such as apoptosis, differentiation, development, proliferation and metabolism [19, 20]. According to previous studies, miRNAs play essential roles in human diseases, including OP [21, 22]. Among these, miR-203-3p could inhibit osteoblast differentiation and promote OP progression [23]. Additionally, Yin et al. elaborated that circ_0076694 could sponge miR-203 and enhance RUNX2 expression to prevent OP [24]. These results indicate that miR-203-3p is modulated by some upstream non-coding RNAs, and plays a repressive role in OP.

It is widely accepted that circRNAs can exert their role as competing endogenous RNAs (ceRNAs) by sponging miRNAs to modulate the pathogenesis of various diseases [25]. This ceRNA network is closely related to OP progression [26]. Based on the above findings, whether circ_0027885 could act as a ceRNA for miR-203-3p to regulate OP has not been studied well. Hence, this work was undertaken to investigate the effects and potential mechanisms of circ_0027885, miR-203-3p and RUNX2 in osteogenesis.

## Materials and methods

### Sample collection

Blood samples were obtained from 22 OP patients aged 43-75 (12 males and 10 females) and 15 normal patients aged 41‐78 (9 males and 6 females) between March 2021 and February 2022. None have complications that influence bone metabolism, such as kidney, liver and blood disease. The clinical study was authorized by the Ethics Committee of Nanjing Lishui People’s Hospital (approval no. 2021SQ25; Nanjing, China). The Declaration of Helsinki was followed for the conduct of this study. All participants were aware of this research and gave their informed consent.

### Cell culture and treatment

Human BMSCs (hBMSCs) were obtained from BeNa Culture Collection (BNCC, JiangSu, China), and incubated in α-MEM with 10% FBS‐HI, 1% penicillin and streptomycin. Next, the cells were plated in T25 flasks at a density of 1 × 105 cells/cm2 in a 5% incubator at 37 °C. The medium was replaced every 2 days. For osteoblast differentiation, the cells at approximately 75‐85% confluency were induced by supplementation with 1% FBS‐HI, 50 µg/ml ascorbic acid, 10 mM β‐glycerophosphate and 0.1 µg/ml dexamethasone. Moreover, the osteogenic media was changed every 3 days.

### Cell transfection

The lentivirus system (LV-NC, LV-circ_0027885, short hairpin (sh) -circ_0027885, sh-NC) was used to infect hBMSCs. These products were purchased from Shanghai Genechem. LV-NC, LV-circ_0027885, sh-NC and sh-circ_0027885 transfected hBMSCs were harvested after incubation with 1 mg/ml puromycin for 72 h. NC mimics, miR-203-3p mimics, NC inhibitor and miR-203-3p inhibitor were obtained from Shanghai GenePharma. At about 70% confluence, hBMSCs were transfected with NC mimics, miR-203-3p mimics, NC inhibitor and miR-203-3p inhibitor by Lipofectamine™2000 (Invitrogen) to perform follow-up experiments.

### Quantitative real-time PCR

Total RNA was extracted by using TRIzol reagent (Invitrogen), and cDNA synthesis was conducted by a commercially available kit (Takara) following the manufacturer’s protocol. RT-qPCR was conducted utilizing SYBR Green (Takara) on the ABI 7500 sequence detection system (Thermo Fisher Scientific). The procedure was set at 95 °C for 15 s and 60 °C for 60 s for a total of 40 cycles. The relative expression was calculated by the comparative (2‐∆∆Ct) method [27]. β‐actin served as a housekeeping gene for circ_0027885 and RUNX2, and U6 served as a housekeeping gene for miR‐203‐3p. The primers used in this study are listed in Table 1.

**Table 1:** Primers used for RT-PCR analysis

Gene	Forward primer	Reverse primer
circ_0027885	5’-AGCCATCTGTGTTGCTGTTG‐3’	5’-ACAAATAAGTTTCCTTGGATACACC‐3’
RUNX2	5’-CCGGTCTCCTTCCAGAAT‐3’	5’-GGGAACTGCTGTGGCTTC‐3’
β-actin	5’-CCATGTACGTTGCTATCCAG‐3’	5’-CTTCATGAGGTAGTCAGTCAG‐3’
miR-203-3p	5’-ATGGTTCGTGGGGTGAAATGTTTAGGAC‐3’	5’-GCAGGGTCCGAGGTATTC‐3’
U6	5’-CTCGCTTCGGCAGCACA‐3’	5’-AACGCTTCACGAATTTGCGT‐3’

### Western blotting

Total cellular proteins were dissolved in radioimmunoprecipitation assay buffer and quantitated in accordance with the BCA protein assay kit protocol (Nanjing KeyGen BioTECH). Protein samples (30 µg) were separated by 10% SDS-PAGE for electrophoresis and transferred to PVDF membranes. Subsequently, the membranes were probed with primary antibodies against RUNX2 (1:1000, #12,556, Cell Signaling Technology, USA) and β‐actin (1:1000, #4970, Cell Signaling Technology, USA) at 4 °C overnight. The next day, the membranes were probed with goat anti‐rabbit IgG (H + L) horseradish peroxidase (HRP)‐conjugated secondary antibody (1:5,000, #14,708, Cell Signaling Technology, USA) for 2 h at room temperature. Finally, the bands were visualized by Quantity One software v4.62 (Bio‐Rad Laboratories).

### Bioinformatics analysis

CircInteractome (https://circinteractome.irp.nia.nih.gov/index.html) was used to predict the potential binding sites between miR-203‐3p and circ_0027885. TargetScan Human v7.2 (http://www.targetscan.org/) was used to predict the targeted binding sites between miR‐203‐3p and RUNX2.

### Dual-luciferase reporter assay

Circ_0027885 wild-type (wt), circ_0027885 mutant (mut), RUNX2 wt and RUNX2 mut sequences were inserted into the pmirGLO plasmids (Promega). 293T cells were cotransfected with the above plasmids and either miR‐203‐3p mimic or mimic‐NC using LipofectamineTM 2000 (Invitrogen). After 2 days, we assayed luciferase activities by the dual‐luciferase assay system (Promega) following the instrument.

**Alkaline phosphatase (ALP) measurement**hBMSCs were harvested and rinsed with PBS for three times. The activities of ALP were detected in cell suspensions using p-nitrophenyl phosphate as a substrate. hBMSCs were suspended in Tris-HCl (Sigma-Aldrich) buffer containing 1% Triton X-100, and then cell lysates were incubated with p-nitrophenyl phosphate buffer. After 10 min of culture at 37 °C, the ALP activities of hBMSCs were measured at 405 nm.

### Alizarin red staining

hBMSCs induced for two-week osteogenic differentiation were washed with pre-cold PBS for three times, and fixed in 70% chilled ethanol for 1 h. After dying in 1% ARS staining solution (Sigma-Aldrich) for 30 min, visible mineralized nodules were captured via an inverted fluorescence microscope (Olympus Corporation).

### Statistical analysis

All experiments were conducted 3 times individually. Alizarin Red staining results were quantified by ImageJ v1.8.0 (National Institutes of Health). Data were expressed as the mean ± standard deviation (SD). Statistical analysis was carried out using GraphPad Prism 8 (GraphPad Software). Student’s t test was used for the comparisons between two groups. One-way ANOVA followed by Tukey’s post hoc test was employed for comparisons among more than two groups. *P* < 0.05 was considered to be significant.

## Results

### Circ_0027885 was induced in osteogenic differentiation

**Fig. 1:**
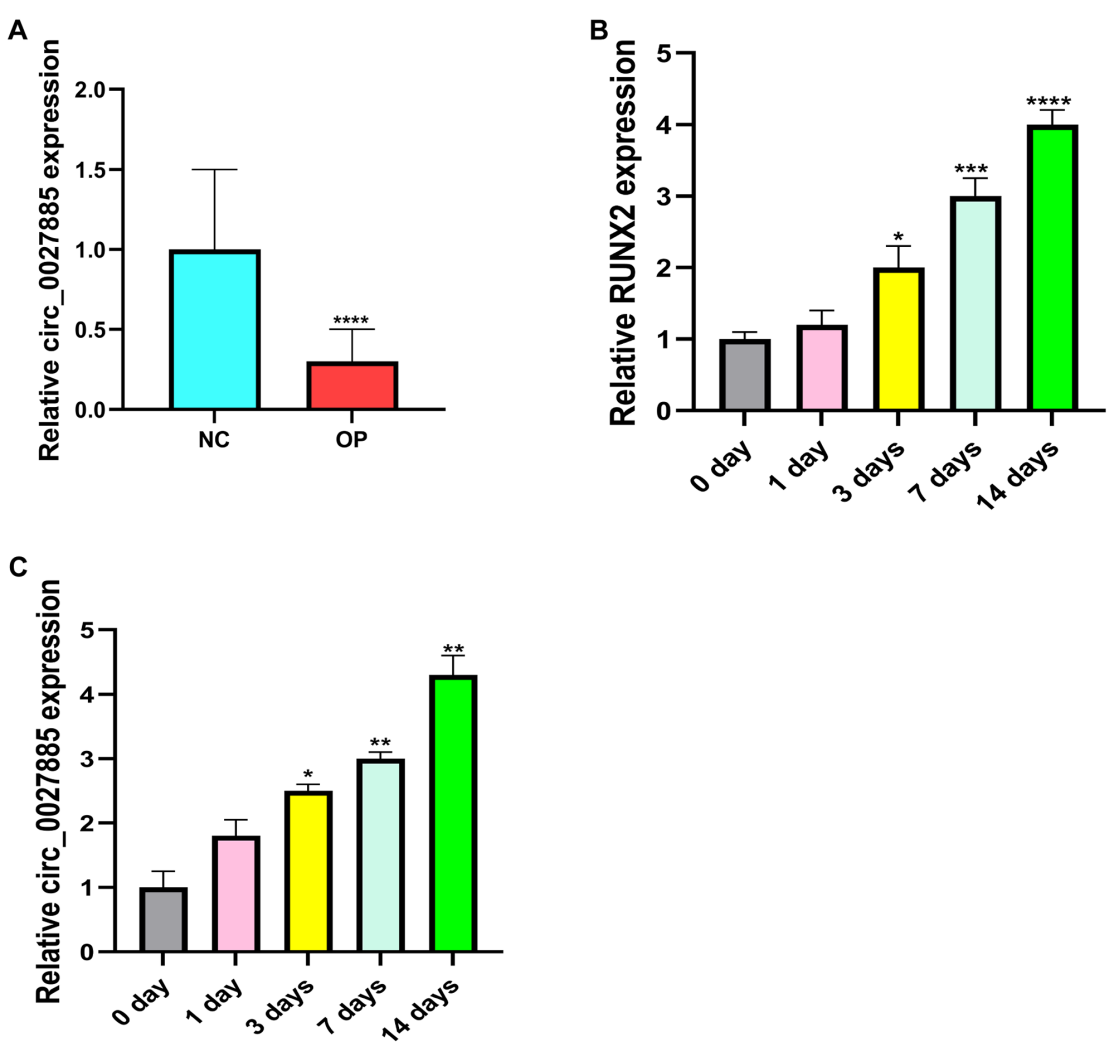
Circ_0027885 was induced in osteogenic differentiation. (**A**) Expression levels of circ_0027885 in normal and OP patients, examined by RT-qPCR. ****P < 0.0001, as comparison to the NC group. (**B-C**) Expression levels of RUNX2 and circ_0027885 in hBMSCs treated with osteogenic differentiation medium for 0, 1, 3, 7, and 14 days, measured by RT-qPCR. *P < 0.05, **P < 0.01, ***P < 0.001, ****P < 0.0001, as comparison to the 0 day group

In the RT-qPCR analysis, circ_0027885 mRNA expression was saliently decreased in serum samples from the OP group compared with the NC group (Fig. 1A). Subsequently, hBMSCs were exposed to osteogenic induction for 0, 1, 3, 7 and 14 days. As shown in Fig. 1B and C, RUNX2 mRNA expression was gradually increased, while circ_0027885 mRNA expression was also dramatically increased in a time-dependent manner. Collectively, circ_0027885 may be involved in the occurrence and progression of OP.

### Circ_0027885 promoted osteogenic differentiation of hBMSCs

**Fig. 2:**
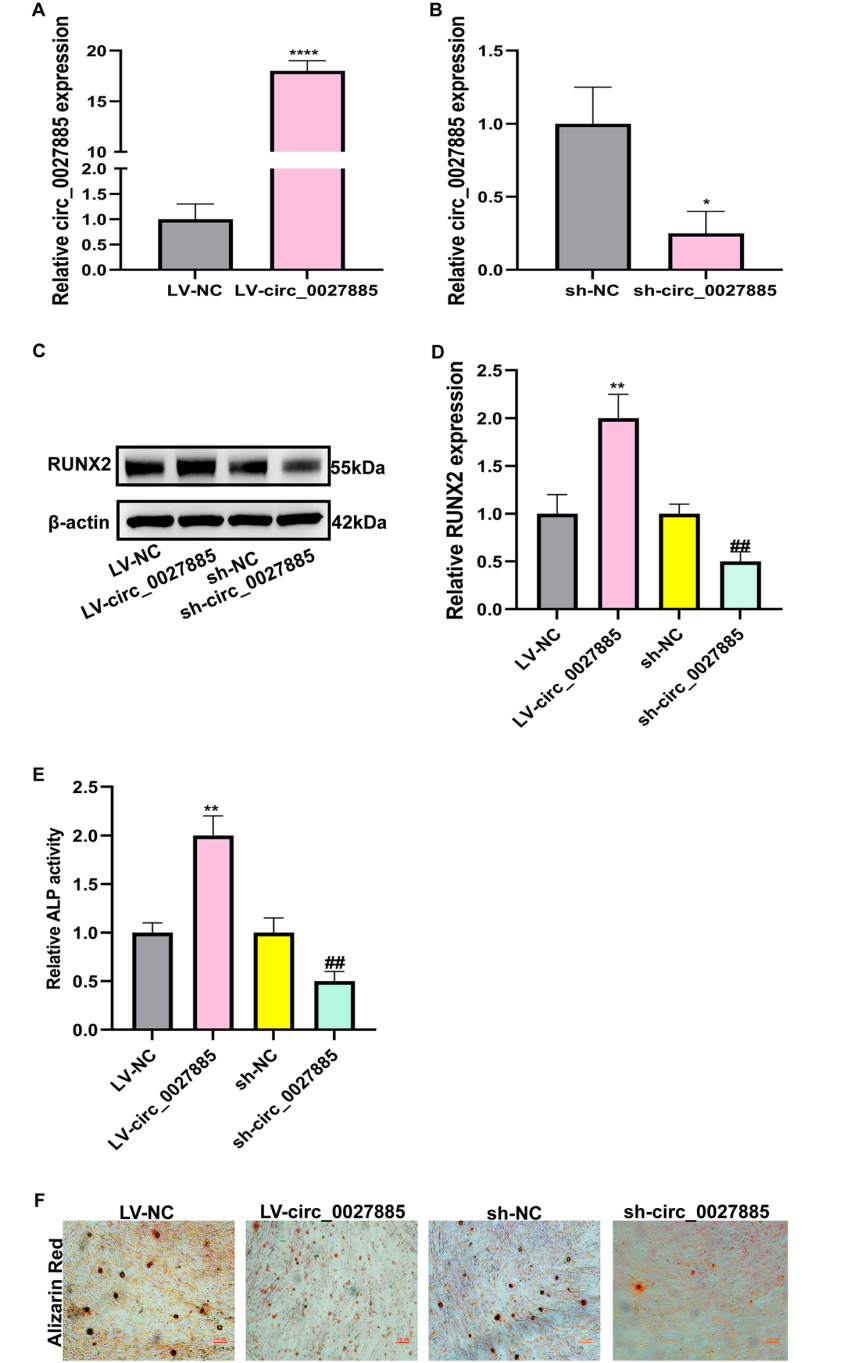
Circ_0027885 promoted osteogenic differentiation of hBMSCs. (**A-B**) Transfection efficiency after infecting LV-circ_0027885 or sh-circ_0027885, validated by qRT-PCR. ****P < 0.0001, as comparison to the LV-NC group, *P < 0.05, as comparison to the sh-NC group. The circ_0027885 expression of LV-NC or sh-NC group was normalized. (**C-D**) Relative protein expression of RUNX2 in hBMSCs after infection with LV-circ_0027885 or sh-circ_0027885, measured by western blot. **P < 0.01, as comparison to the LV-NC group, **P < 0.01, as comparison to the sh-NC group. The RUNX2 expression of LV-NC group was normalized. (**E**) Relative ALP activity was examined in hBMSCs after infection with LV-circ_0027885 or sh-circ_0027885. **P < 0.01, as comparison to the LV-NC group, **P < 0.01, as comparison to sh-NC group. The ALP activity of LV-NC group was normalized. (**F**) The mineralization levels of hBMSCs after infection with LV-circ_0027885 or sh-circ_0027885, as determined by Alizarin red staining

To identify the biological function of circ_0027885 on OP, RT-qPCR analysis was applied to check the efficiency of the transfection in the LV-circ_0027885 and sh‐circ_0027885 groups. Compared to the LV-NC group, the expression of circ_0027885 was significantly enhanced in the LV-circ_0027885 group (Fig. 2A), but markedly decreased in the sh‐circ_0027885 group (Fig. 2B). On day 14 after osteogenic induction in hBMSCs, western blot analysis showed that RUNX2 protein levels were upregulated in the LV-circ_0027885 group and downregulated in the sh‐circ_0027885 group (Fig. 2C-D). The activity of ALP, which is closely related to osteogenic differentiation, was increased by overexpression of circ_0027885, and silencing circ_0027885 reduced ALP activity in hBMSCs (Fig. 2E). To observe the mineralized matrix affected by circ_0027885, the results of Alizarin Red staining showed that the osteogenic differentiation levels were increased in the LV-circ_0027885 group and decreased in the sh‐circ_0027885 group (Fig. 2F). In summary, we speculated that circ_0027885 could facilitate the osteogenic differentiation of hBMSCs.

### MiR-203‐3p was sponged by circ_0027885

**Fig. 3:**
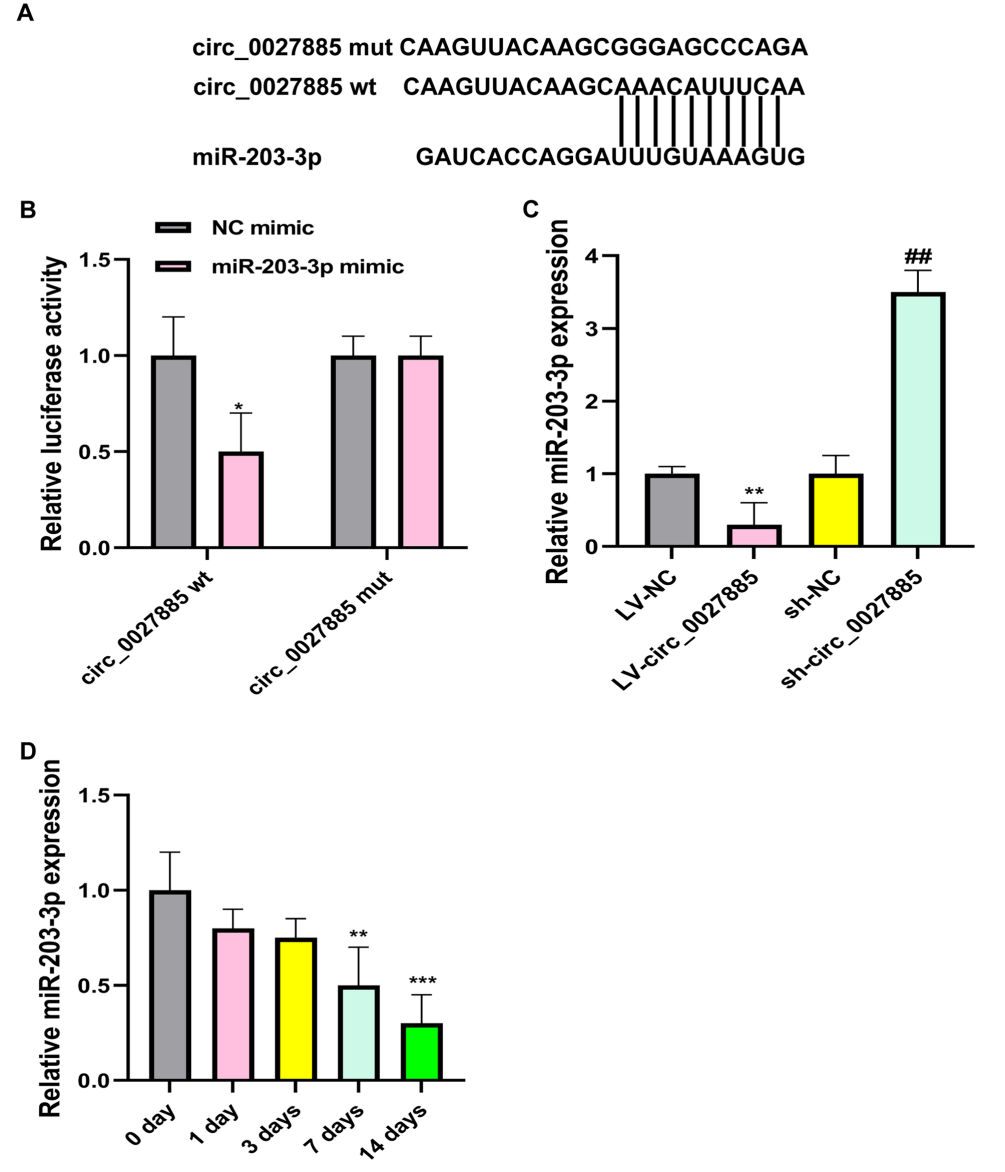
MiR-203‐3p was sponged by circ_0027885. (**A**) The binding sites between miR‐203‐3p and circ_0027885, predicted by bioinformatics analysis. (**B**) Circ_0027885-wt and circ_0027885-mut luciferase activity in 293T cells treated with miR‐203‐3p mimic or NC mimic, determined by dual‐luciferase reporter gene assay. *P < 0.05, as comparison to the NC mimic group. The luciferase activity of circ_0027885-wt with NC mimic group was normalized. (**C**) Relative expression of miR-203-3p in hBMSCs after infection with LV-circ_0027885 or sh-circ_0027885, validated by qRT-PCR. **P < 0.01, as comparison to the LV-NC group, **P < 0.01, as comparison to the sh-NC group. The miR-203-3p expression of LV-NC group was normalized. (**D**) Relative expression of miR‐203‐3p in hBMSCs treated with osteogenic differentiation medium for 0, 1, 3, 7, and 14 days, detected by RT-qPCR. **P < 0.01, ***P < 0.001, as comparison to the 0 day group. The miR-203-3p expression of 0 day group was normalized

As indicated previously, circRNAs act as miRNA sponges to modulate molecular mechanisms, and we predicted the circ_0027885 interacting miRNA by bioinformatics analysis. Applying CircInteractome software, we identified that circ_0027885 has potential binding sites for miR-203‐3p (Fig. 3A). In addition, dual‐luciferase reporter gene assay manifested that the miR‐203‐3p mimic markedly downregulated luciferase activity in the wild-type circ_0027885 group, but exhibited no significant effect in the mutant-type circ_0027885 group (Fig. 3B). Overexpression of circ_0027885 resulted in the decreased expression of miR‐203‐3p, while knockdown of circ_0027885 showed the reverse result (Fig. 3C). Moreover, dramatically reduced miR‐203‐3p expression was observed in a time-dependent manner during osteogenic differentiation (Fig. 3D). Generally, miR‐203‐3p could act as a meaningful target of circ_0027885.

### MiR-203‐3p inhibited osteogenic differentiation of hBMSCs

**Fig. 4:**
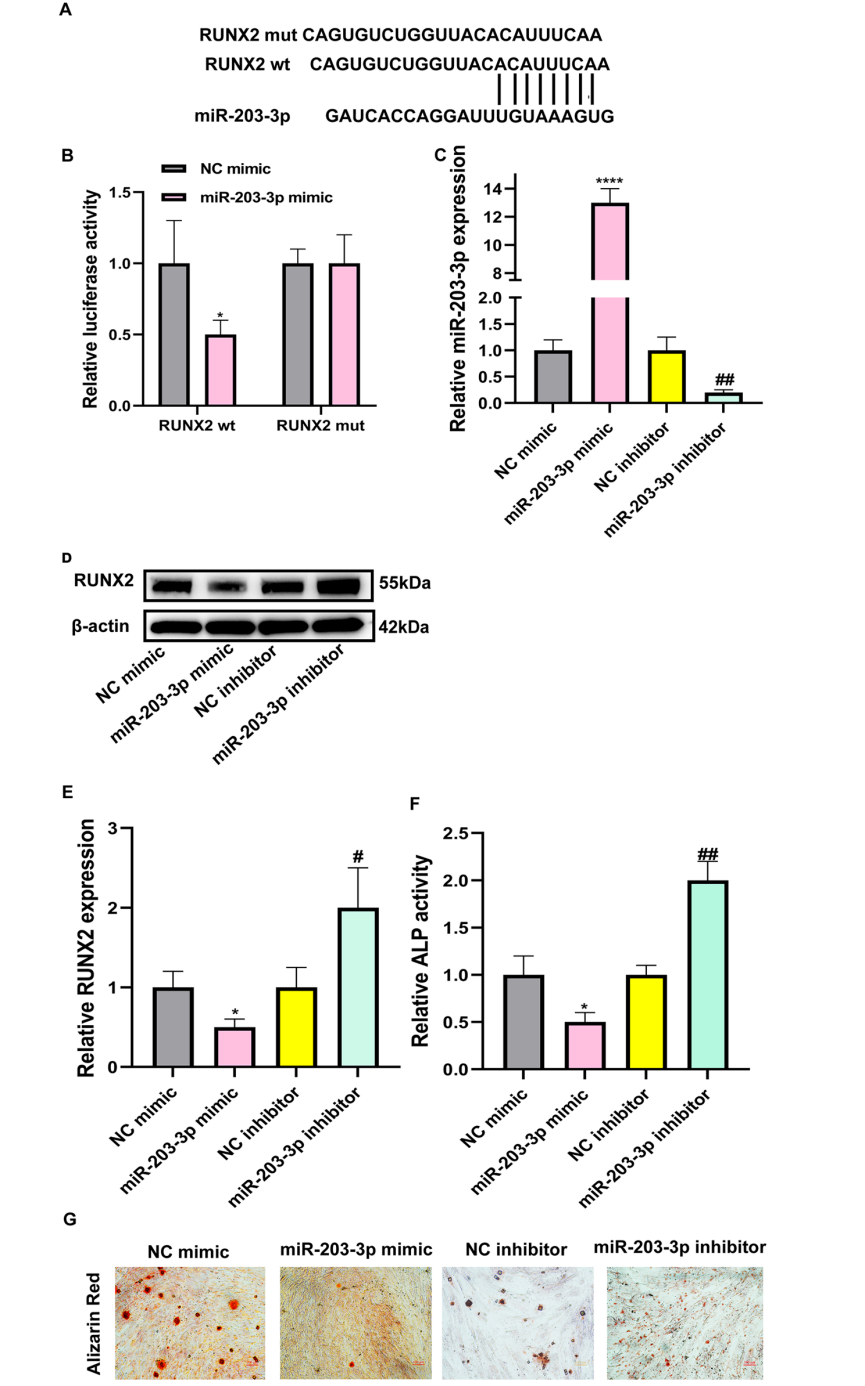
MiR-203‐3p inhibited osteogenic differentiation of hBMSCs. (**A**) The binding sites between miR‐203‐3p and RUNX2, predicted by bioinformatics analysis. (**B**) RUNX2-wt and RUNX2-mut luciferase activity in 293T cells treated with miR‐203‐3p mimic or NC mimic, determined by dual‐luciferase reporter gene assay. *P < 0.05, as comparison to the NC mimic group. The luciferase activity of RUNX2-wt with NC mimic group was normalized. (**C**) Transfection efficiency after coculturing miR‐203‐3p mimic or miR‐203‐3p inhibitor, validated by qRT-PCR. ****P < 0.0001, as comparison to the NC mimic group; **P < 0.01, as comparison to the NC inhibitor group. The miR-203-3p expression of NC mimic group was normalized. (**D-E**) Relative protein expression of RUNX2 in hBMSCs after transfection with miR‐203‐3p mimic or miR‐203‐3p inhibitor, measured by western blot. *P < 0.05, as comparison to the NC mimic group; *P < 0.05, as comparison to the NC inhibitor group. The RUNX2 expression of NC mimic group was normalized. (**F**) Relative ALP activity was examined in hBMSCs after transfecting miR‐203‐3p mimic or miR‐203‐3p inhibitor. *P < 0.05, as comparison to the NC mimic group; **P < 0.01, as comparison to the NC inhibitor group. The ALP activity of NC mimic group was normalized. (**G**) The mineralization levels of hBMSCs after transfecting miR‐203‐3p mimic or miR‐203‐3p inhibitor, employed by Alizarin red staining

As further predicted by TargetScan online bioinformatics software, RUNX2 was selected as a potential downstream candidate of miR-203‐3p (Fig. 4A). The results of dual‐luciferase reporter gene assay verified that relative luciferase activity was notably reduced after the cotransfection of miR‐203‐3p mimic and wild-type RUNX2, but had no effect on the mutant-type RUNX2 (Fig. 4B). To demonstrate the functions of miR‐203‐3p on the osteogenic differentiation of hBMSCs, we transfected miR‐203‐3p mimic and miR‐203‐3p inhibitor. As displayed in Fig. 4C, miR‐203‐3p level was highly expressed in miR‐203‐3p‐overexpressing hBMSCs, but expressed at low levels in miR‐203‐3p-silenced hBMSCs. Western blot analysis showed that the protein level of RUNX2 was significantly decreased after treatment with the miR‐203‐3p mimics and partially recovered after treatment with the miR‐203‐3p inhibitor (Fig. 4D-E). ALP activity was blocked by miR-203‐3p overexpression, while silencing miR-203-3p increased ALP activity in hBMSCs (Fig. 4F). Alizarin Red staining revealed that miR‐203‐3p mimics remarkably inhibited hBMSCs mineralization ability, whereas miR‐203‐3p inhibitor reversed the changes (Fig. 4G). Taken together, miR‐203‐3p could regulate RUNX2 expression in hBMSCs.

### Circ_0027885 regulated hBMSCs osteogenic differentiation via miR-203‐3p/RUNX2 axis

**Fig. 5:**
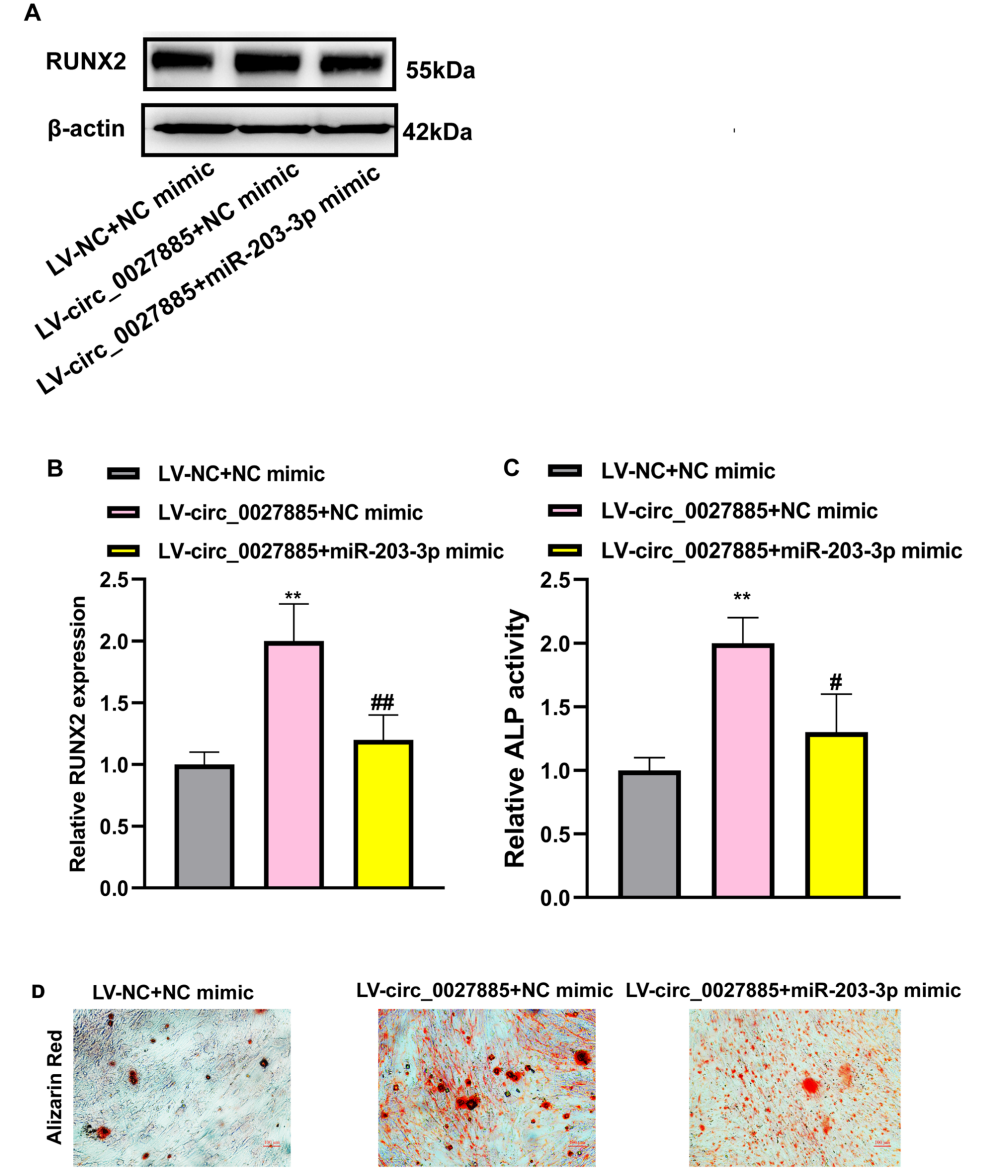
Circ_0027885 regulated hBMSCs osteogenic differentiation via the miR-203‐3p/RUNX2 axis. (**A-B**) Relative protein expression of RUNX2 in LV-circ_0027885 + NC mimic or LV-circ_0027885 + miR‐203‐3p mimic treated hBMSCs, measured by western blot. **P < 0.05, as comparison to the LV-NC + NC mimic group; **P < 0.01, as comparison to the LV-circ_0027885 + NC mimic group. (**C**) Relative ALP activity was examined in LV-circ_0027885 + NC mimic or LV-circ_0027885 + miR‐203‐3p mimic treated hBMSCs. **P < 0.01, as comparison to the LV-NC + NC mimic group; *P < 0.05, as comparison to the LV-circ_0027885 + NC mimic group. (**D**) The mineralization levels in LV-circ_0027885 + NC mimic or LV-circ_0027885 + miR‐203‐3p mimic treated hBMSCs, as determined by Alizarin red staining

Circ_0027885 silencing upregulated miR-203‐3p levels, and downregulated RUNX2 levels in hBMSCs. Simultaneously, it was confirmed that miR‐203‐3p was the upstream miRNA of RUNX2. Therefore, it was urgent to explore whether circ_0027885 regulates RUNX2 levels by sponging miR‐203‐3p. We found that circ_0027885 overexpression considerably upregulated RUNX2 protein levels, while cotransfection of miR‐203‐3p mimics partially reversed this change (Fig. 5A-B). In addition, ALP activity was increased by circ_0027885 overexpression, which was partially abated following transfection with miR-203-3p mimics (Fig. 5C). Moreover, Alizarin Red staining further revealed that cotransfection of miR‐203‐3p mimics reversed the formation of maximal nodules in hBMSCs (Fig. 5D). It could be concluded that circ_0027885 regulated osteogenic differentiation of hBMSCs through the miR‐203‐3p/RUNX2 axis.

## Discussion

Osteoporosis is a systemic metabolic bone disorder that often leads to the progression of fragility fractures [28]. It is mostly caused by dysregulation of bone remodeling due to the interaction between genetic and environmental factors, dietary habits, and lifestyle [29]. The balance of bone formation and resorption and the maintenance of bone homeostasis in bone remodeling are closely related. Hence, it is critical to investigate the osteogenic pathogenesis and molecular mechanism for OP treatment. Herein, we excitedly identified circ_0027885, which has the potential to alleviate OP progression.

Up to now, much interest has been focused on the role of circRNA in osteoblast differentiation. They can exert essential effects on a variety of physiological and pathological processes [30]. For instance, hsa_circ_0001275 could function as a putative diagnostic biomarker for postmenopausal OP [31]. Circ_0002060 was validated to be a key therapeutic target in OP(28). A clinical research has identified that circ_0027885 levels are notably decreased in OP patients [32]. However, there are no studies on the function and mechanism of circ_0027885 in OP. This work revealed that circ_0027885 was reduced in OP patients. Then, we carried out overexpression and knockdown experiments to further discover the effect of circ_0027885 in OP. The analysis suggested that circ_0027885 could take part in the osteogenic differentiation of hBMSCs, which was confirmed by RUNX2 expression and Alizarin Red staining. Hence, we proposed that circ_0027885 is vital to the bone formation and osteogenic differentiation of hBMSCs.

CircRNAs are not only directly involved in regulating the development of OP, but also act as ceRNAs that competitively bind miRNAs to regulate downstream target genes, thus affecting the pathogenesis of OP [33]. Wang et al. revealed that circRNA_0006393 promoted osteogenesis in glucocorticoid-induced BMSC osteogenic differentiation by regulating the miR‐145‐5p/FOXO1 pathway [34]. Yu et al. reported that circRNA_0016624 could facilitate postmenopausal osteoporosis by sponging miR-98 and enhancing BMP2 expression [35]. As predicted by bioinformatics analyses, miR‐203‐3p was identified to bind circ_0027885. Subsequently, our work focused on the miR‐203‐3p level during hBMSCs osteogenic differentiation and its association with circ_0027885. These above findings demonstrated that miR‐203‐3p was declined after stimulating osteogenic differentiation and negatively regulated by circ_0027885. Additionally, miR‐203‐3p was confirmed to have a binding site for RUNX2, and its overexpression could reduce RUNX2 expression during osteogenesis of hBMSCs.

RUNX2 is widely known as one of the most important transcription factors in the early stages of osteoblast differentiation [36, 37]. According to the results in this study, overexpressed circ_0027885 could upregulate the protein level of RUNX2 to promote hBMSCs osteogenic differentiation. Moreover, overexpressed miR-203‐3p could remarkably reduce the protein level of RUNX2. After overexpression of circ_0027885 and miR‐203‐3p, the protein expression levels exhibited the opposite results compared with the LV-circ_0027885 + NC mimic group. The present results were in line with those of Alizarin Red staining. Above all, these findings validated that circ_0027885 regulated RUNX2 by sponging miR‐203‐3p as a ceRNA in OP.

It should be noted that this study has some limitations. First, it was conducted in vitro using human bone marrow mesenchymal stem cells (hBMSCs), which may not fully reflect the complex pathophysiology of osteoporosis in vivo. In our future studies, we will verify the conclusions of this paper through animal experiments. Additionally, the sample size used in this study was relatively small, which may limit the generalizability of the findings. Further research with larger sample sizes and animal models is needed to confirm these results and explore their clinical implications.

## Conclusion

**Fig. 6:**
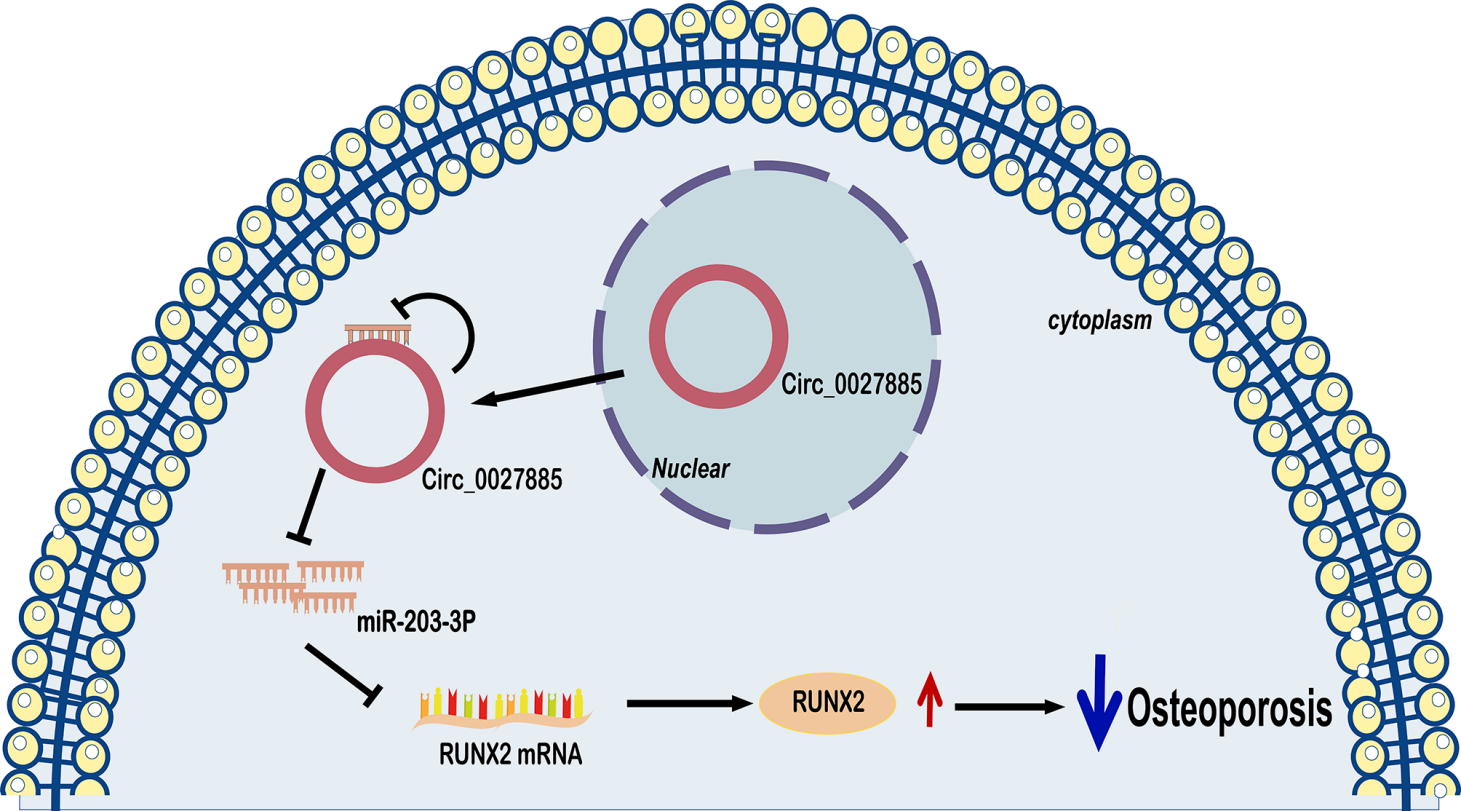
The circ_0027885/miR-203‐3p/RUNX2 axis is a novel key pathway in OP progression

In summary, this work is the first to determine the new molecular mechanism of the circ_0027885/miR-203‐3p/RUNX2 pathway in OP progression. More specifically, circ_0027885 suppressed miR‐203‐3p expression by sponging miR‐203‐3p and enhanced RUNX2 expression to promote osteogenesis in hBMSCs (Fig. 6).

## Data Availability

The analyzed data sets generated during the study are available from the corresponding author on reasonable request.
